# Vision-based particle filtering for quad-copter attitude estimation using multirate delayed measurements

**DOI:** 10.3389/frobt.2023.1090174

**Published:** 2023-05-30

**Authors:** Nargess Sadeghzadeh-Nokhodberiz, Mohammad Iranshahi, Allahyar Montazeri

**Affiliations:** ^1^ Electrical and Computer Engineering Department, Qom University of Technology, Qom, Iran; ^2^ Engineering Department, Lancaster University, Lancaster, United Kingdom

**Keywords:** UAV, quad-copter, particle filtering, multi-rate sensor fusion, attitude estimation, camera, gyroscope (gyro)

## Abstract

In this paper, the problem of attitude estimation of a quad-copter system equipped with a multi-rate camera and gyroscope sensors is addressed through extension of a sampling importance re-sampling (SIR) particle filter (PF). Attitude measurement sensors, such as cameras, usually suffer from a slow sampling rate and processing time delay compared to inertial sensors, such as gyroscopes. A discretized attitude kinematics in Euler angles is employed where the gyroscope noisy measurements are considered the model input, leading to a stochastic uncertain system model. Then, a multi-rate delayed PF is proposed so that when no camera measurement is available, the sampling part is performed only. In this case, the delayed camera measurements are used for weight computation and re-sampling. Finally, the efficiency of the proposed method is demonstrated through both numerical simulation and experimental work on the DJI Tello quad-copter system. The images captured by the camera are processed using the ORB feature extraction method and the homography method in Python-OpenCV, which is used to calculate the rotation matrix from the Tello’s image frames.

## 1 Introduction

Autonomous quad-copter UAVs are increasingly employed in various industries, especially in applications with extreme environments where humans cannot access narrow, high altitude, far reaching, and confined spaces for further operation and inspection (Montazeri et al., 2021). Of particular importance is the ability of quad-copters to accurately maneuver in hazardous and unstructured environments such as those existing in the nuclear decommissioning applications. One of the challenging tasks for navigation of drones in such GPS-denied environments is finding the exact position and orientation of the quad-copters for feedback control and characterization of the environment (Burrell et al., 2018). Nowadays, the inertial navigation system (INS) including inertial measurement units (IMU) is widely used for navigation of UAVs. Toward this, first of all, a robust and reliable attitude estimator is required which should be able to execute on low-cost computational hardware and using measurements from light-weight sensors (Bassolillo et al., 2022).

Attitude estimation is the procedure of estimating orientation of the vehicle with respect to a reference frame using sensory measurements such as inertial and attitude sensors. Although least square error (LSE) and maximum likelihood (ML) approaches can be classified as early attitude estimation methods, model-based Bayesian approaches are most common and precise approaches can be found in Dhahbane et al. (2021). Model-based approaches normally employ vehicle kinematics and/or dynamics to provide a prediction from the orientation, and the predicted attitude is updated through the sensory measurements (Sadeghzadeh-Nokhodberiz and Poshtan, 2016; Ozaki and Kuroda, 2021). There are an increasing number of research studies devoted to attitude estimation (Moutinho et al., 2015; Nokhodberiz et al., 2019; Liang, 2017). The commonly used stochastic approaches are the Kalman filter (KF) and extended Kalman filter (EKF) (Nemati and Montazeri, 2019). However, in KF-based methods (KF, EKF, and UKF), only Gaussian noise processes are considered and EKF suffers from the linearization issue. Therefore, in some research studies, particle filters (PF) are used to overcome the problem in attitude estimation of UAVs (Cheng and Crassidis, 2004; Sadeghzadeh-Nokhodberiz et al., 2014b). The gyroscope measurements in the body frame are normally incorporated in the attitude kinematics to obtain the orientation in the inertial frame. The gyroscope noises can be modeled through a probability distribution function, making the kinematics a stochastic model as it is employed as an input vector in it. Therefore, it is necessary to employ a stochastic approach such as PF that works directly with the non-linear dynamic model of the system. PFs are appropriate for attitude estimation of quad-copters due to non-linear and non-stochastic nature of the system model. PF is an optimal non-linear filtering method in which the posterior probability density function (pdf) is approximated through sample point (particles) generation as it is not possible to be computed analytically for non-Gaussian systems. This posterior pdf is required for Bayesian minimum mean square error (MMSE) estimation, and it is the main advantage of PF over other non-linear Bayesian MMSE estimators such as EKF (Sadeghzadeh-Nokhodberiz and Meskin, 2020).

Additional sensors such as cameras are commonly employed together with the low-cost inertial sensors. This greatly helps mitigate the effect of errors and noises in the gyroscope measurement and facilitates designing a vision-based navigation technique (Sadeghzadeh-Nokhodberiz et al., 2014a; Sadeghzadeh-Nokhodberiz et al., 2014c). Although cameras can provide highly accurate measurements from the quad-copter orientation compared to low-cost gyroscopes, they suffer from a slow sampling rate and delay problems with respect to the gyroscope measurements due to heavy computation load required. In the vision-based navigation, feature points extracted from the camera images are tracked and the camera motion, mounted on the UAV, is related to the locations of tracked planar feature points in the image plane using the homography relationship (Wang et al., 2013; White and Beard, 2019). Homography-based state estimation of a quad-copter system using EKF is presented in Chavez et al. (2017). The images captured by cameras should be highly processed for feature extraction including detection, description, and matching (Csurka et al., 2018). Although a recently developed ORB (Rublee et al., 2011) method can significantly reduce the processing time compared to the popular SIFT (Lowe, 2004) and SURF (Bay et al., 2006) approaches, it still needs almost 33 m for feature extraction per image (Mur-Artal et al., 2015). This processing time not only leads to a much slower sampling rate but also the measured values are received with a significant delay for the attitude estimation procedure.

The problem of multi-rate delayed state estimation has been studied in Lin and Sun (2021), Comellini et al. (2020), Fatehi and Huang (2017), and Khosravian et al. (2015). Lin and Sun (2021) and Khosravian et al. (2015) proposed a cascaded output predictor and an attitude observer where the effect of sampling and delays are compensated in the predictor. The delayed measurements are extrapolated to present time using past and present estimates of the KF in Larsen et al. (1998), where an optimal gain is derived for this extrapolated measurement. In Lin and Sun (2021), the system with delayed and multi-rate measurements is transformed into a delay-free and single-rate system using a state iterating method, and a non-augmented recursive optimal linear state filter is presented for the system by utilizing projection theory. In Fatehi and Huang (2017), different KFs are employed for each type of measurement and the estimates are fused considering the correlation between them in the next step. The cross-covariance matrix between the estimation errors of KFs is obtained iteratively to be employed in the fusion process. Due to the non-linear attitude kinematics with respect to the Euler angles and its stochastic nature due to the incorporation of the gyroscope noise in the model, the PF is an appropriate choice for the attitude estimation. It is worth mentioning that as long as staying away from singularity points (±90 deg rotations of pitch angle), the Euler angle representation of the attitude is preferred to the quaternion representation as the quaternion must obey its normalization constraint, which can cause issues in the filtering (Markley and Crassidis, 2014). In Bassolillo et al. (2022), a KF-based sensor fusion algorithm, using a low-cost navigation platform that contains an inertial measurement unit (IMU), five ultrasonic ranging sensors, and an optical flow camera is proposed to improve navigation of a UAV system in indoor GPS-denied environments. A multi-rate version of the EKF is employed to deal with the use of heterogeneous sensors with different sampling rates and the presence of non-linearity in the model.

To the best of the authors’ knowledge, the problem of PF-based attitude estimation using PF with multi-rate delayed sensors has not yet been studied in the literature. Accordingly, in this paper, a multi-rate delayed PF is proposed to estimate the orientation with a discretized attitude kinematics in Euler angles. It is shown that the corresponding weights of the generated particles are the likelihood of generally non-Gaussian delayed camera measurements. The result is then validated through simulation and experiments on a UAV quad-copter system. For the experimental work, a DJI Tello quad-copter system is employed where the images are processes using the ORB feature extraction method and Python-OpenCV is employed to calculate the rotation matrix using the homography approach.

The organization of the paper proceeds as follows. The system and measurement models including the attitude kinematic model, and gyroscope and camera measurement models are presented in Section 2. Section 3 provides with the PF with multi-rate delayed measurements. Simulation results are presented in Section 4 to demonstrate the accuracy of the presented PF. The experimental data gathered from DJI Tello quad-copter systems are analyzed in Section 5. Finally, conclusion is provided in Section 6.

## 2 System and measurement models

### 2.1 System model

The quad-copter attitude kinematics which represents the relationship between angular velocities in the body and inertial frames are described as follows Sadeghzadeh-Nokhodberiz et al. (2021):

$$\begin{array}{cc} & \dot{\phi }\left(t\right)=p\left(t\right)+\sin\left(\phi \left(t\right)\right)\tan\left(\theta \left(t\right)\right)q\left(t\right)+\cos\left(\phi \left(t\right)\right)\tan\left(\theta \left(t\right)\right)r\left(t\right),\\ & \dot{\theta }\left(t\right)=\cos\left(\theta \left(t\right)\right)q\left(t\right)-\sin\left(\phi \left(t\right)\right)r\left(t\right),\\ & \dot{\psi }\left(t\right)=\frac{\sin\left(\phi \left(t\right)\right)}{\cos\left(\theta \left(t\right)\right)}q\left(t\right)+\frac{\cos\left(\phi \left(t\right)\right)}{\cos\left(\theta \left(t\right)\right)}r\left(t\right), \end{array}$$ (1)

where ***x*** (*t*) = [*ϕ*(*t*) *θ*(*t*) *ψ*(*t*)]^*T*^ is the attitude vector of quad-copter which is defined in the inertial frame where roll angle *ϕ*(*t*), pitch angle *θ*(*t*), and yaw angle *ψ*(*t*) determine rotations around *x*-axis, *y*-axis, and *z*-axis, respectively. In addition, *p*(*t*), *q*(*t*), and *r*(*t*) are angular velocities rotating around *x*-axis, *y*-axis, and *z*-axis in the body frame, respectively, and *t* refers to time.

### 2.2 Measurement models

#### 2.2.1 The gyroscope measurement model

The gyroscope measurement model can be written as follows:

$$\omega_{m}\left(k\right)=\omega_{b}\left(k\right)+\nu_{\omega }\left(k\right),$$ (2)

where $\omega_{m}\left(k\right)\in R^{3}$ and $\omega_{b}\left(k\right)\in R^{3}$ are the vectors of measured and true angular velocities in the body frame at sample time *k* and ***ω***_*b*_ = [*p q r*]^*T*^ (see Eq. 1). Moreover, $\nu_{\omega }\in R^{3}$ is a zero-mean generally non-Gaussian measurement noise with a known probability density function (pdf) with the covariance matrix of ***R***_*ω*_.

#### 2.2.2 The camera measurement model

**FIGURE 1:**
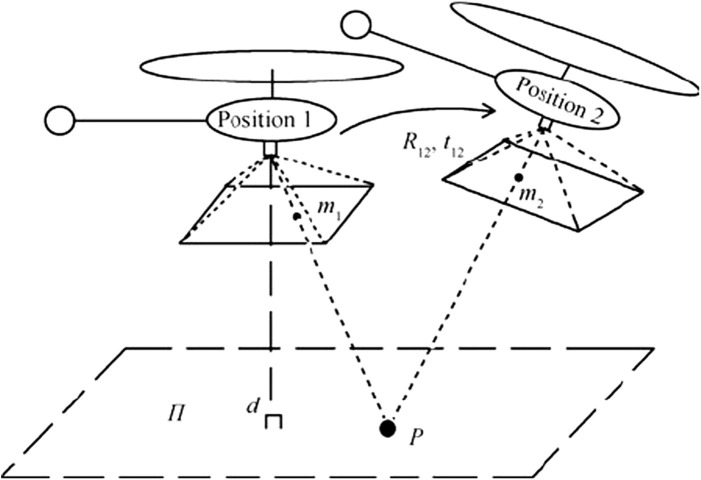
Same fixed point, *P*, viewed from two different positions of a moving quad-copter for the homography purpose Wang et al. (2013).

Homography is used to obtain the measurement model of camera by providing the transformation (including scale, rotation, and translation) between two images. Toward this, two consecutive frames from a camera mounted on a moving body viewing a fixed point *P* are considered. The fixed point is considered a feature extracted from the images using some feature extraction approaches such as ORB (Rublee et al., 2011).

singular value decomposition (SVD) is then performed with the feature pairs that pass the RANSAC test to calculate the homography matrix *H*. Let *m*_1_ and *m*_2_ be the two projections of point *P* in the camera coordinates with *R*_12_ and *t*_12_ as the corresponding rotation matrix and translation vector, respectively, in the camera frame transforming *m*_1_ to *m*_2_ (see Figure 1). In Figure 1, *d* is the Euclidean distance between the plane *π*, with the unit normal vector *n*, and position 1. The relationships between the homography matrix and the transformation between two images can be found in Wang et al. (2013). Finally, through the SVD of the homography matrix, *R*_12_ and *t*_12_ can be obtained which can be transformed to the direction cosine matrix (DCM), *R*, iteratively. The Euler angles can be then computed using the DCM.

Therefore, the camera measurement model without delay consideration can be represented as

$$y_{e}\left(k\right)=x\left(k\right)+\nu_{e}\left(k\right),$$ (3)

where ***ν***_*e*_ is a zero-mean generally non-Gaussian measurement noise with the covariance matrix of ***R***_*e*_. It is worth mentioning that the index *e* refers to the Euler angles.

## 3 The proposed multirate particle filter

In this section, a PF for the system and measurement models introduced in the previous section is presented when the sensors collect data using a multi-rate sampling frequency procedure.

### 3.1 The general model

**FIGURE 2:**
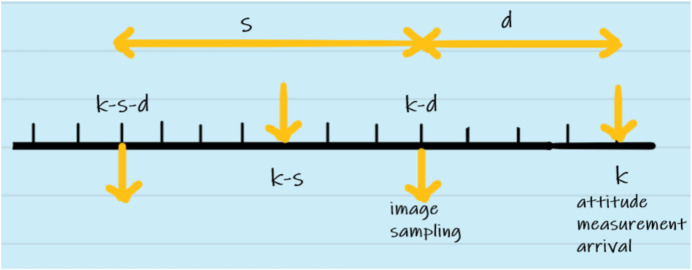
Camera sampling and delay scenario.

Toward this, let the gyroscope and camera sampling times in seconds be represented with *T* and *sT*, respectively, with s∈N . Moreover, we consider the camera processing delay time as *dT* with d∈N and *d* < *s*, as depicted in Figure 2.

Moreover, a discretized form of the kinematic model presented in Eq. 1 is also considered with the general non-linear discrete state space model as follows:

$$x_{k}=f\left(x_{k-1},u_{k}\right)+\varpi_{k-1},$$ (4)

where ***u***_*k*_ = [*p*(*k*)*q*(*k*)*r*(*k*)]^*T*^ and ***x***_*k*_ is defined in Equation 1 and ***ϖ***_*k*−1_ is the additive process noise resulting from the gyroscope measurement noise ***ν***_*ω*_(*k*) with the same distribution but a scaled covariance matrix ***Q***_*x*_. The index *k* refers to the sampling instant.

In other words, the components of the discretized model of (4) are as follows:

$$\begin{array}{cc} f\left(x_{k-1},u_{k}\right) & =x_{k-1}+TA_{k-1}u_{k},\\ \varpi_{k-1} & =TA_{k-1}\nu_{\omega }\left(k\right), \end{array}$$ (5)

where $A_{k-1}=\left[\begin{array}{ccc} 1 & \sin\left(\phi_{k-1}\right)\tan\left(\theta_{k-1}\right) & \sin\left(\phi_{k-1}\right)\tan\left(\theta_{k-1}\right)\\ 0 & \cos\left(\theta_{k-1}\right) & \cos\left(\theta_{k-1}\right)\\ 0 & \frac{\sin\left(\phi_{k-1}\right)}{\cos\left(\theta_{k-1}\right)} & \frac{\cos\left(\phi_{k-1}\right)}{\cos\left(\theta_{k-1}\right).} \end{array}\right]$ .

### 3.2 The modified particle filter

The general approach in the PF is to compute the posterior pdf using the Monte Carlo (MC) method used in the Bayesian estimation of the stochastic process ***x***_*k*_ by having the measurement history ***y***_1:*k*_ = {***y***_1_, … , ***y***_*k*_} and the current sample of input at time *k*. The goal in standard PF is to approximate the posterior pdf *p* (***x***_*k*_| ***y***_1:*k*_) by generating particles from a known distribution and estimating the target pdf through attribution of the normalized weights for each particle.

However, in case of this study as explained earlier, the measurements are delayed and a slower sampling rate is considered for the camera compared with the data measured from the gyroscope. Therefore, two different cases may happen at each sampling step. The delayed camera measurements are arrived or there are no camera measurements. In this section, the PF for these two cases are derived.

#### 3.2.1 Case I: camera measurement available

In this case, as depicted in Figure 2, the delayed camera measurement is available at sample time *k*. Therefore, the posterior distribution *p* (***x***_1:*k*_| ***y***_*e*,1:*s*:*k*−*d*_) should be approximated using the MC method such that ***y***_*e*,1:*s*:*k*−*d*_ refers to the historical data collected by camera every *s* sampling instant and with the initial time delay of *d*. Here, the proposal distribution *p* (***x***_*k*_| ***x***_*k*−1_) is employed for the particle generation. By applying the Bayes’ rule and the Markov property, it can be concluded that

$$\begin{array}{cc} p\left(x_{1:k}|y_{e,1:s:k-d}\right) & =p\left(x_{k}|x_{k-1}\right)\times \ldots \times p\left(x_{k-d+1}|x_{k-d}\right)\\ & \;\times p\left(x_{1:k-d}|y_{e,1:s:k-d}\right). \end{array}$$ (6)

Here, *p* (***x***_1:*k*−*d*_| ***y***_*e*,1:*s*:*k*−*d*_) using the Bayes’ rule and statistical independencies can be rewritten as follows:

$$\begin{array}{cc} p & \left(x_{1:k-d}|y_{e,1:s:k-d}\right)\propto p\left(y_{e,k-d}|x_{k-d}\right)\times p\left(x_{1:k-d}|y_{e,1:s:k-d-s}\right). \end{array}$$ (7)

The term *p* (***x***_1:*k*−*d*_| ***y***_*e*,1:*s*:*k*−*d*−*s*_) is also extended as follows:

$$\begin{array}{cc} p\left(x_{1:k-d}|y_{e,1:s:k-d-s}\right)= & \;p\left(x_{k-d}|x_{k-d-1}\right)\times \ldots \times p\left(x_{k-s+1}|x_{k-s}\right)\\ & \times p\left(x_{1:k-s}|y_{e,1:s:k-d-s}\right). \end{array}$$ (8)

Using Eqs 6–8, the weight function is computed as follows:

$$w\left(x_{k}\right)=p\left(y_{e,k-d}|x_{k-d}\right)w\left(x_{k-s}\right).$$ (9)

The weight functions are evaluated for the particles generated using the proposal distribution *p* (***x***_*k*_| ***x***_*k*−1_) and using the system probabilistic model represented by the kinematics model in Eq. 1. So the particles $x_{k}^{i-}{|}_{i=1}^{N}$ are generated where *N* refers to the number of particles. Since re-sampling should be carried out as the next step, the weights of the particles at sample time *k* − *s* are transformed to $\frac{1}{N}$ after re-sampling with re-sampled particles of $x_{k-s}^{i+}{|}_{i=1}^{N}$ .

Therefore, the normalized weights for re-sampling for each particle are computed as follows:

$$w_{k}^{i*}=\frac{p\left(y_{e,k-d}|x_{k-d}^{i+}\right)}{\overset{N}{\underset{j=1}{\sum }}p\left(y_{e,k-d}|x_{k-d}^{j+}\right)}.$$ (10)

#### 3.2.2 No camera measurement available

In this case, no camera data are available at sample time *k*. Therefore, *p* (***x***_1:*k*_| ***y***_*e*,1:*s*:*k*−*d*−*s*_) should be approximated. Therefore, the weight function is *w* (***x***_*k*_) = *w* (***x***_*k*−*s*_) as it is proved in the following. Since the last received measurement from the camera at the sampling instant *k* is ***y***_*k*−*d*−*s*_ and received at *k* − *s* sampling instant, therefore

$$\begin{array}{cc} p\left(x_{1:k}|y_{e,1:s:k-d-s}\right)= & \;p\left(x_{k}|x_{k-1}\right)\times \ldots \times p\left(x_{k-d+1}|x_{k-d}\right)\\ & \;\times p\left(x_{1:k-d}|y_{e,1:s:k-d-s}\right), \end{array}$$ (11)

where the term *p* (***x***_1:*k*−*d*_| ***y***_*e*,1:*s*:*k*−*d*−*s*_) is computed in Eq. 8 which states that the corresponding weight of the *i* ^*th*^ particle $x_{k-1}^{i-}$ is $w\left(x_{k-s}^{i+}\right)$ and after normalization and re-sampling the corresponding weight of each particle would become $\frac{1}{N}$ .

In order to clarify the proposed method, it is presented in Pseudo code in Table 1.

**TABLE 1:** Pseudo code corresponding to the proposed PF for attitude estimation.

**Step 0**: *Initialization*: Sample initial particles, that is, ${\left\{x_{0}^{i}\right\}}_{i=1}^{N}$ , using a known initial distributions of states (*p* (*x*_0_)), where *x*_0_ ∼ *p* (*x*_0_)
At the time instant *k*
**Step 1**: *Prior estimate*: Generate the prior state particles ${\left\{x_{k}^{i-}\right\}}_{i=1}^{N}$ using the system model, that is, $x_{k}^{i-}∼p\left(x_{k}|x_{k-1}^{i+}\right)$ , for *i* = 1: *N*
In other words for the attitude system of (4) and 5: $x_{k}^{i-}=x_{k-1}^{i+}+TA_{k-1}^{i+}\omega_{m}\left(k\right)-\varpi_{k-1}^{i},$ where $x_{k}^{i-}$ is the prior estimate at the time sample *k* and $x_{k-1}^{i+}$ is the re-sampled posterior estimate at the time sample *k* − 1 and $A_{k-1}^{i+}=A_{k-1}{|}_{x_{k-1}^{i+}}$ and $\varpi_{k-1}^{i}$ is the particle generated using a known pdf of ***ϖ***_*k*−1_ which is zero mean generally non-Gaussian noise with the covariance of $Q_{x}=T^{2}A_{k-1}^{i+}R_{\omega }{\left(A_{k-1}^{i+}\right)}^{T}$
**Step 2**: *Posterior estimate*: Compute ${\left\{x_{k}^{i+}\right\}}_{i=1}^{N}$ as follows.
**IF** the camera measurement, ***y***_*e*,*k*−*d*_, is available, the weights are computed using (10) and the particles are re-sampled to generate posterior estimates with equal weights of $\frac{1}{N}$ , that is, ${\left\{x_{k}^{i+},\frac{1}{N}\right\}}_{i=1}^{N}$
**ELSE** let $x_{k}^{i+}=x_{k}^{i-},\;i=1,.,N$ with the corresponding weights of $\frac{1}{N}$ for each sample
**END**
**Step 3**: *State estimation*: Estimate the system states as ${\hat{x}}_{k}=\sum_{i=1}^{N}\frac{1}{N}x_{k}^{i+}$

## 4 Simulation results

In this section, simulation results are provided in the MATLAB/SIMULINK environment to show the efficiency of the proposed method. The simulated AR Drone Parrot 2.0 quad-copter is stabilized using a non-linear robust sliding mode control technique presented in Nemati and Montazeri (2018a) and Nemati and Montazeri (2018b).The physical parameters of the quad-copter are listed as follows:

$$\;I_{xx}=7.72\times 10^{-2}kg\;m^{2},\;I_{yy}=7.64\times 10^{-2}kg\;m^{2},$$

$$I_{zz}=0.1031kg\;m^{2},\;I_{r}=1.8\times 10^{-5}kg\;m^{2},\;m=2.5kg.$$

**FIGURE 3:**
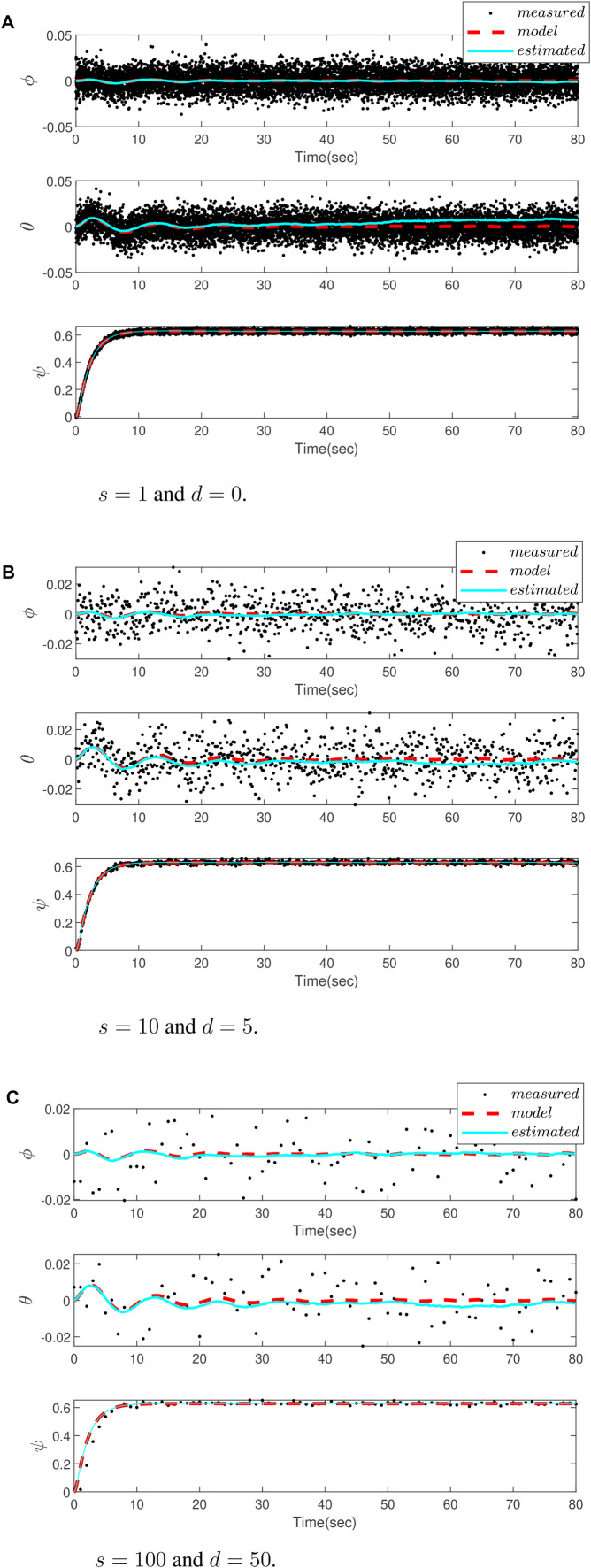
Comparing attitude estimation of a drone for a different delay *d* and sampling time *s*, using measurement by a camera (dots), estimation by the proposed particle filter (blue dot-line), and the kinematic model (red dot-line). **(A)** No delay and sampling rate for both gyro and camera **(B)** Sampling rate of the camera is 10 times slower than the gyro and the delay in the camera measurements is 5 samples **(C)** Sampling rate of the camera is 100 times slower than the gyro and the delay in the camera measurements is 50 samples.

In order to evaluate the performance of the proposed PF, different scenarios for delay and sampling rate values are considered. Figure 3A depicts the attitude estimation result using the gyro and camera measurements, and the results are compared with those obtained from the kinematics model when camera has no delay and the sampling rates are equal, that is, *s* = 1 and *d* = 1. Figures 3B,C are related to the cases with *s* = 10, *d* = 5 and *s* = 100, *d* = 50, respectively. It is obvious from the figures that although the camera slows the sampling rate and processing delay, and deteriorates the estimation accuracy, the proposed method is still successful to provide an accurate estimation.The root mean square error (RMSE) criterion is also employed to provide a numerical measure for a comparative study of the results. The results are summarized in Table 2, which also confirms the aforementioned discussion.

**TABLE 2:** RMSE for different scenarios.

Scenario	*s* = 1, *d* = 0	*s* = 10, *d* = 5	*s* = 100, *d* = 50
RMSE	9.7001 × 10^−6^	3.2284 × 10^−5^	8.2284 × 10^−5^

## 5 Experimental results

**FIGURE 4:**
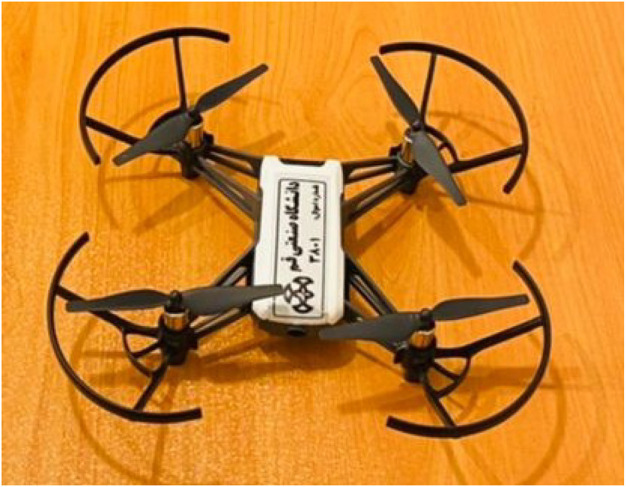
Image of the DJI Tello mini-drone.

The experimental results are provided using the DJI Tello drone illustrated in Figure 4. It is a small (99 *mm* × 92.5 *mm* × 41 *mm*) and lightweight (80 *g*) drone with a maximum speed of 8 *m*/*s* which uses the 2.4 GHz Wi-Fi communication channel to be connected to a PC or laptop for sending and receiving telemetry data and commands, respectively. The drone is equipped with an IMU and a 720 *p* camera and an SDK is provided to help developers for implementation of their algorithms. Although the camera information is available, the SDK cannot read the IMU data and instead the results of internal positioning data can be read Steenbeek (2020).

To perform the homography, first, the ORB feature matching method is applied on the frames captured by the DJI Tello camera. Toward this, the ORB algorithm uses the improved FAST algorithm, used in image feature point detection, the feature point screening, image pyramid building, and the feature point direction determination. After that, the ORB algorithm uses the improved BRIEF algorithm to generate binary feature point descriptors, and then, the descriptors are corrected using the steer BRIEF method to include the direction information. Finally, in the process of feature point matching, the points are matched based on their descriptor similarities. Toward this, the Brute-Force matcher method applied in Hamming distance is used to measure the distance between the binary descriptors and to choose the nearest ones as the matched points. Finally, by employing the PROSAC algorithm, the matched points with larger matching errors are rejected, which significantly improves the accuracy of matching Luo et al. (2019).

For this purpose, in Python-OpenCV, we have employed the following command for the feature point detection and generation of descriptors:

$$\left[keypoints,descriptors\right]=orb.detectAndCompute\left(img\right)$$

**FIGURE 5:**
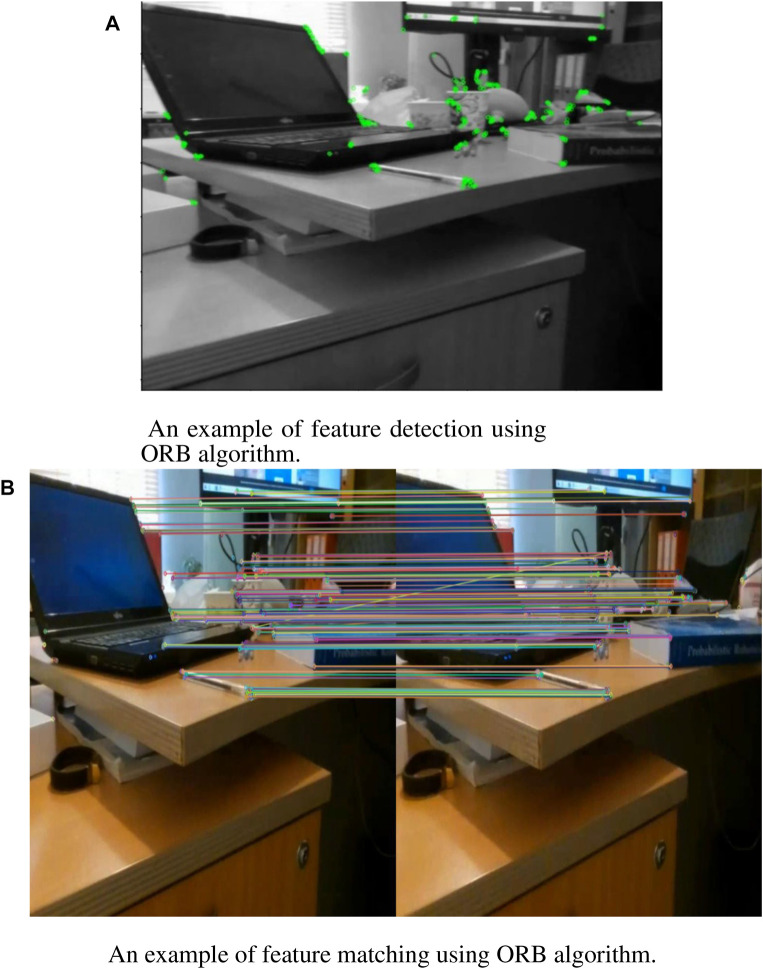
ORB feature extraction results achieved by the drone camera in a hover position. **(A)** Feature extraction **(B)** Feature matching.

The result of this command for a sample frame is depicted in Figure 5A. In addition, for feature matching, the following command is employed:

$$matches=bf.match\left(des1,des2\right)$$

where *des*1 and *des*2 are descriptor vectors of two successive frames. Finally, the matched points are sorted to find the best matches. The result of feature matching is depicted in Figure 5B for the same sample frames.

To compute the homography matrix, the following command is employed:

$$H=cv.findHomography\left(srcPoints,dstPoints\right),$$

where *srcPoints* are coordinates of the points in the original plane, which is a cell array of 2-element vectors {[*x*, *y*], …} with single floating-point precision and *dstPoints* are coordinates of the points in the target plane, of the same size and type as *srcPoints*. Now, in order to compute the DCM from the computed homography matrix, the following command is employed:

$$\left[n,Rs,Ts,Ns\right]=cv2.decomposeHomographyMat\left(H,K\right).$$

Here, *H* is the input homography matrix between two images, *K* is the input camera intrinsic matrix, *Rs* are array of rotation matrices, *Ts* are array of translation matrices, and *Ns* are array of plane normal matrices. In addition, *n* is the number of possible solutions and returned as the function output.The set of four solutions is returned using this command which can be reduced to two or one using the method explained in Malis and Vargas (2007). Reducing the number of solutions to two can be achieved by using additional constraints. For this purpose, a set of reference image points p* is selected and by using the camera intrinsic matrix *K*, the points are projected using the relation as follows:

$$m^{*}=K^{-1}p^{*}.$$

In this case, the valid solutions are those satisfying the projection inequality as follows for all points in the plane determined by the normal vector m* and n* is the normal vector of the corresponding plane

$$m^{*T}n^{*}>0.$$

**FIGURE 6:**
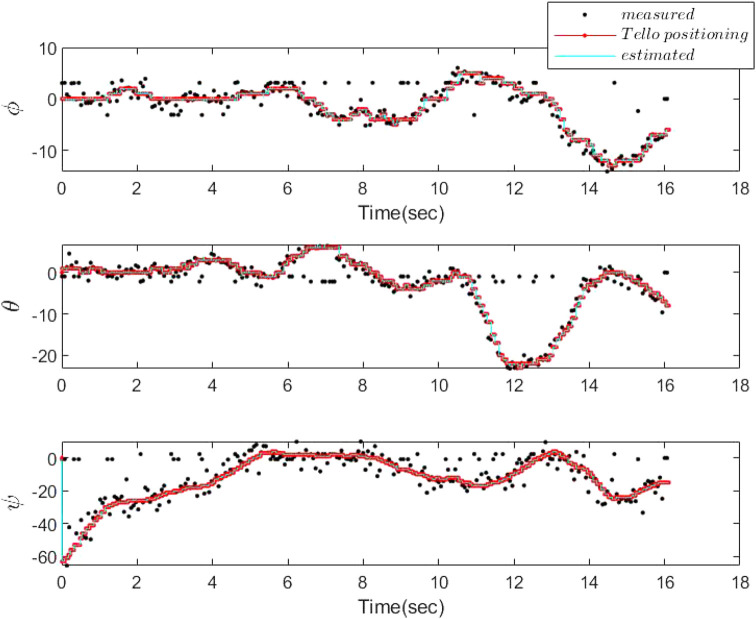
Attitude estimation *versus* the experimental data from the DJI Tello with varying *d* and *s*.

The frame rate of the camera is 30 frames per sec, and we have processed the frames on an 11th Gen Intel(R) Core(TM) i7-1165G7 @ 2.8 GHz processor. The results of the attitude estimation using the proposed PF compared to the camera homography (measurement results) and the positioning results, recorded by DJI Tello, are depicted in Figure 6. It is worth mentioning that due to experimental limitations the sampling time and delay in the recorded experimental data have not been constant, but they are known. However, the delay caused by the camera processing time has always been less than these varying sampling periods. Therefore, the proposed approach is applicable to the problem at hand. In other words, the camera frames are sampled after the results of the ORB and homography procedures of the last sampled frames are available. It took on average 0.03 s for processing of each frame for our processor and 0.02 s for the ORB feature extraction and matching, and the homography procedure only needs 4 × 10^−4^ s. The sampling rate of the positioning procedure on average is 17 × 10^−4^ s. Therefore, on average *d* = 30 and *s* = 40. In other words, the sampling rate of the camera is 0.068 s, and thus, only one frame is missed due to the frame rate of 30 frames per sec. Accordingly, the processing delay of the camera information is less than the sampling time of the camera. Although we have processed the gathered data from the camera offline, it is also possible to be processed online. In other words, sampling two successive frames is fast enough such that still there exists features to be matched between two sample frames (only one frame is missed). Moreover, in practical implementation of the particle filter estimation algorithm, since the IMU data are not directly available for measurement, the gathered positioning data by camera are used for the particle generation. This is replaced by the kinematic model used in the simulation results.

## 6 Conclusion

An extension of the sampling importance re-sampling (SIR) particle filter (PF) was proposed in this paper to solve the problem of attitude estimation of a quad-copter system equipped with a multi-rate camera and gyroscope sensors. In the proposed PF, the delayed camera measurements are used for weight computation and re-sampling and when no camera measurement is available, only the sampling is performed. It was shown through simulation and experimental data that the method is successful to estimate the attitude truly in the presence of delayed multi-rate camera measurements. In the experimental part, the ORB feature matching method was employed for image processing in Python-OpenCv, and after that, the DCM was computed using homography.As our future research topic, we intend to solve the problem of attitude estimation using particle filtering, in the presence of gyroscope faults and errors such as sensory biases and drifts, as well as delayed multi-rate camera measurements.

## Data Availability

The raw data supporting the conclusion of this article will be made available by the authors, without undue reservation.
